# Supplementary material for: Synthesis of New Planar-Chiral Linked [2.2]Paracyclophanes-N-([2.2]-Paracyclophanylcarbamoyl)-4-([2.2]Paracyclophanylcarboxamide, [2.2]Paracyclophanyl-Substituted Triazolthiones and -Substituted Oxadiazoles
Source: Molecules. 2020 Jul 22;25(15):3315. doi: 10.3390/molecules25153315 (PMC7436044; doi:10.3390/molecules25153315)
Supplement: Supplementary file 1 [file molecules-25-03315-s001.pdf]

Supplementary Information for

Synthesis of New Planar-Chiral Linked [2.2]paracyclophanes-*N*-([2.2]-paracyclophanylcarbamoyl)-4-([2.2]paracyclophanylcarboxamide, [2.2]paracyclophanyl-Substituted Triazolthiones and -Substituted Oxadiazoles

**Ashraf A Aly [1,*], Stefan Bräse [2,3,*], Alaa A. Hassan [1], Nasr K. Mohamed [1],
Lamiaa E. Abd El-Haleem [1,2] and Martin Nieger [4]**

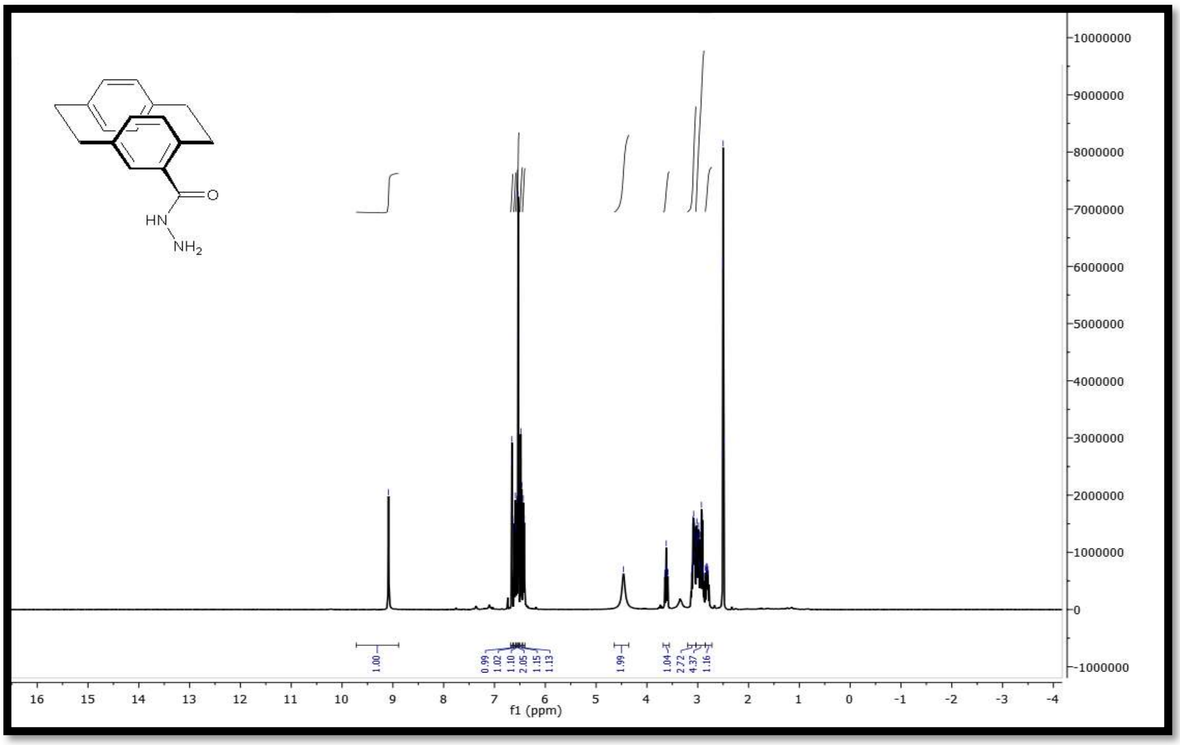

SI Figure 1. ^{1}H NMR of compound (*rac*)-**1**

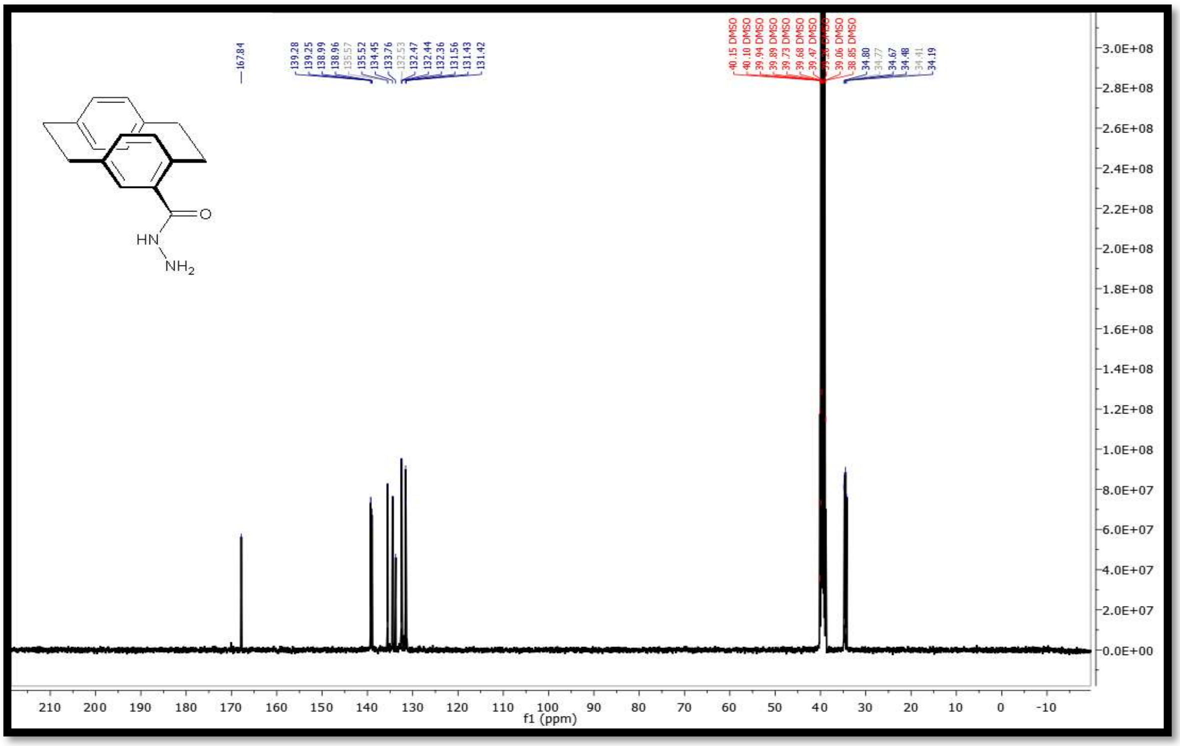

SI Figure 2. ^{13}C NMR of compound (*rac*)-**1**

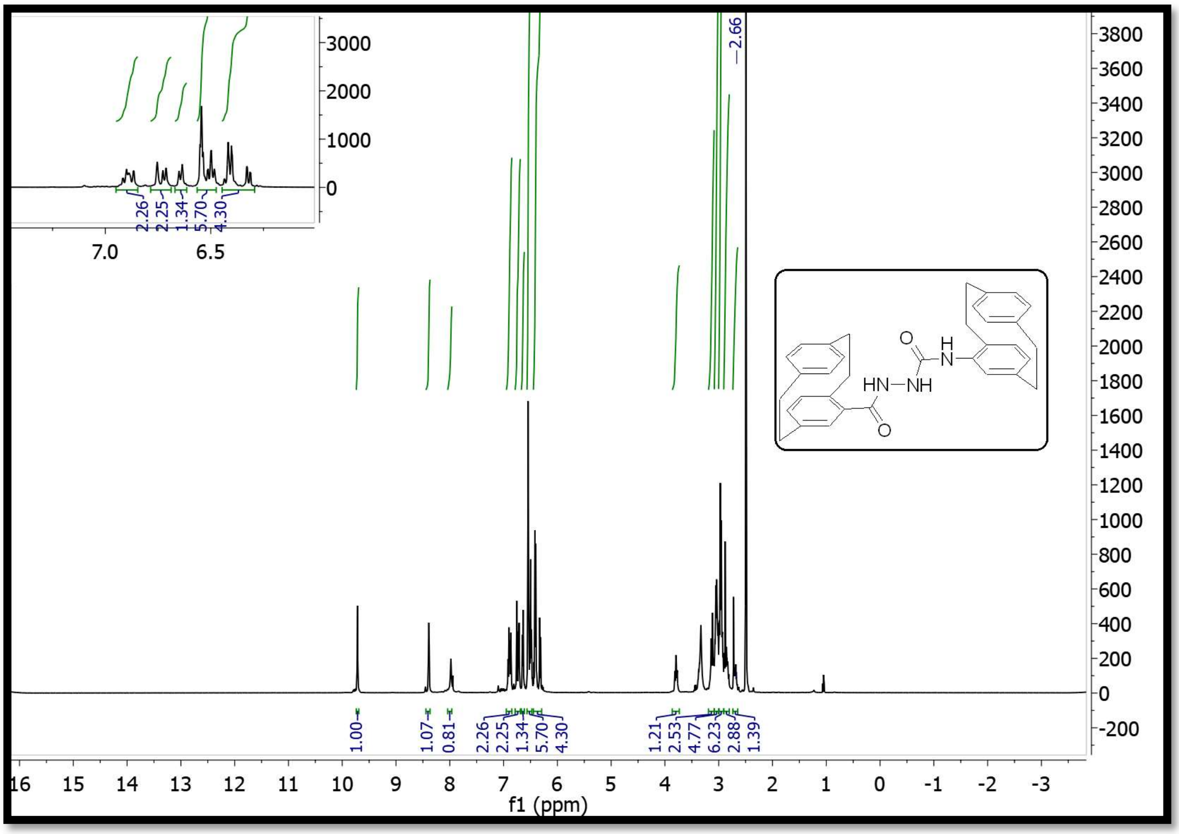

SI Figure 3. ^{1}H NMR of *diastreomer*-**3**

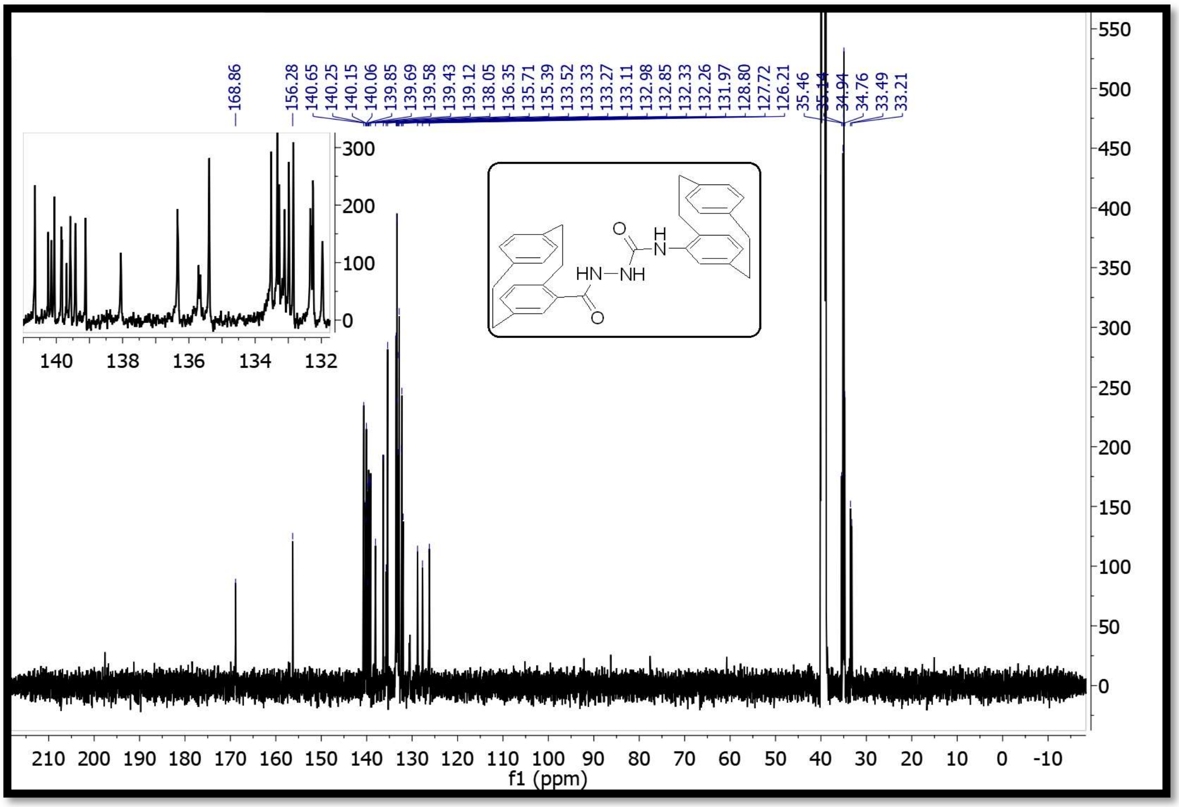

SI Figure 4. ^{13}C NMR of diastreomer-**3**

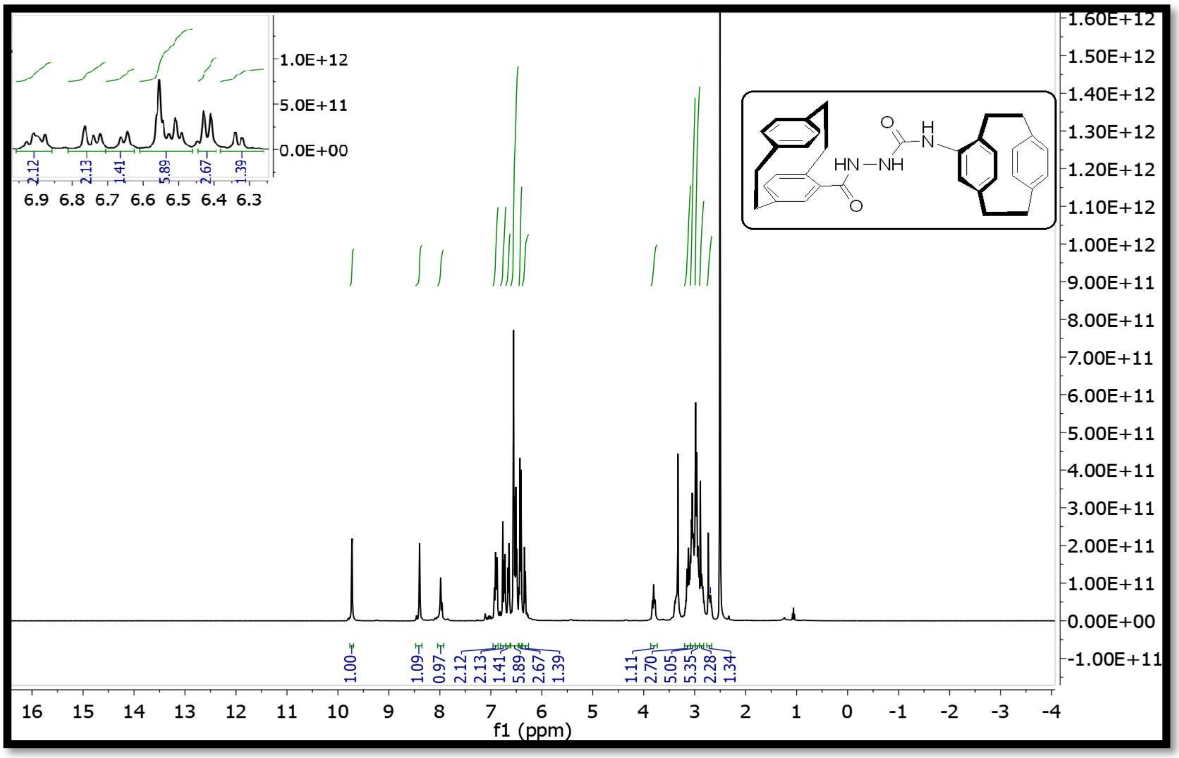

SI Figure 5. ^{1}H NMR spectrum of compound (*Sp*-*Sp*)-**3**

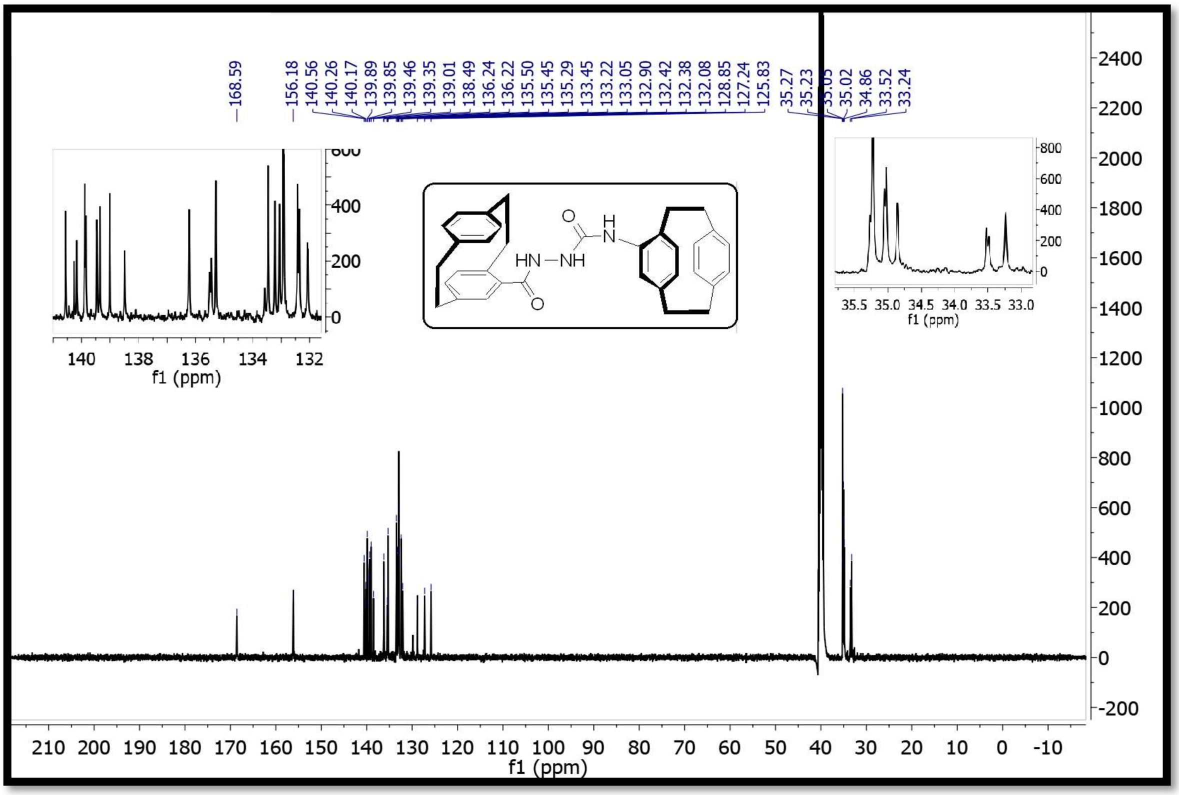

SI Figure 6. ^{13}C NMR spectrum of compound (*Sp*-*Sp*)-**3**

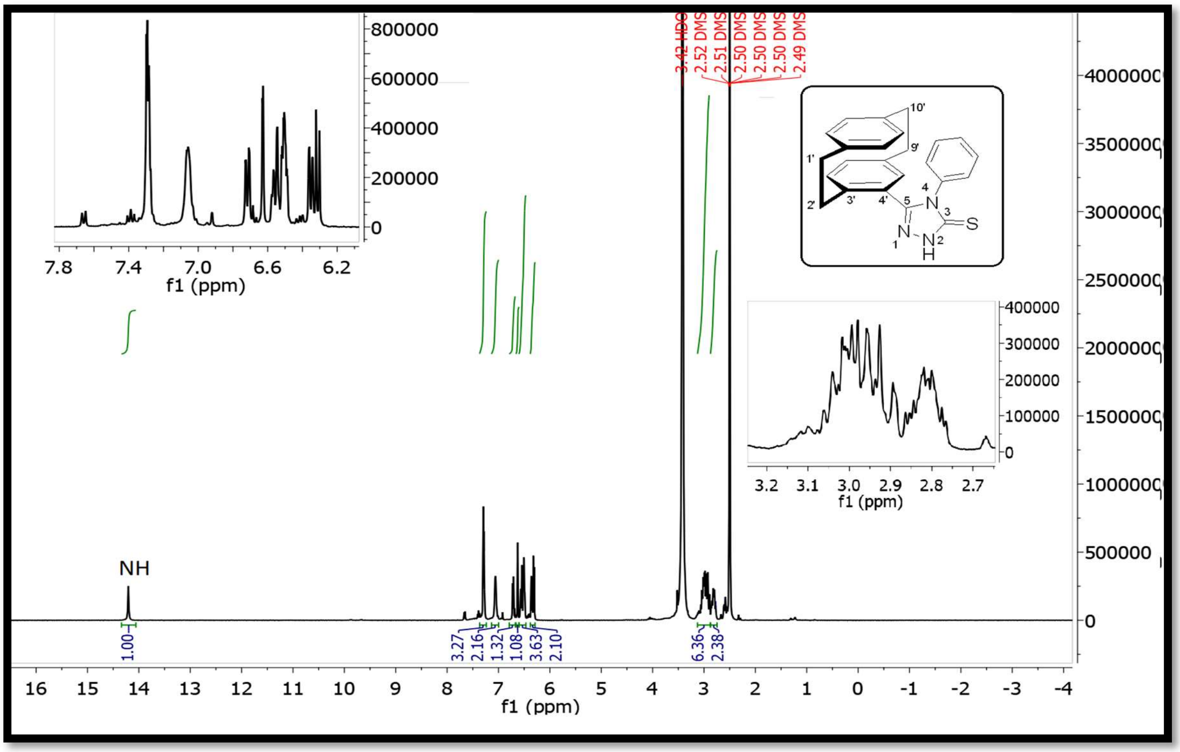

SI Figure 7. ^{1}H NMR of compound **12a**

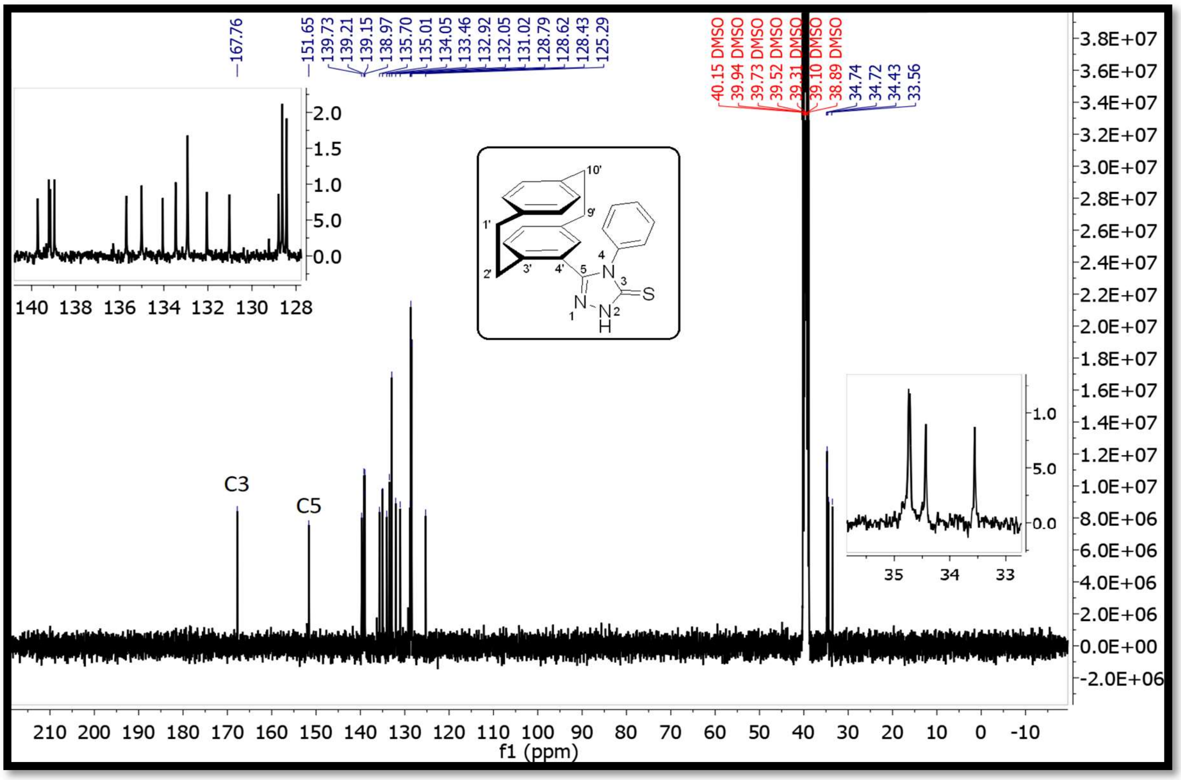

SI Figure 8. ^{13}C NMR of compound **12a**

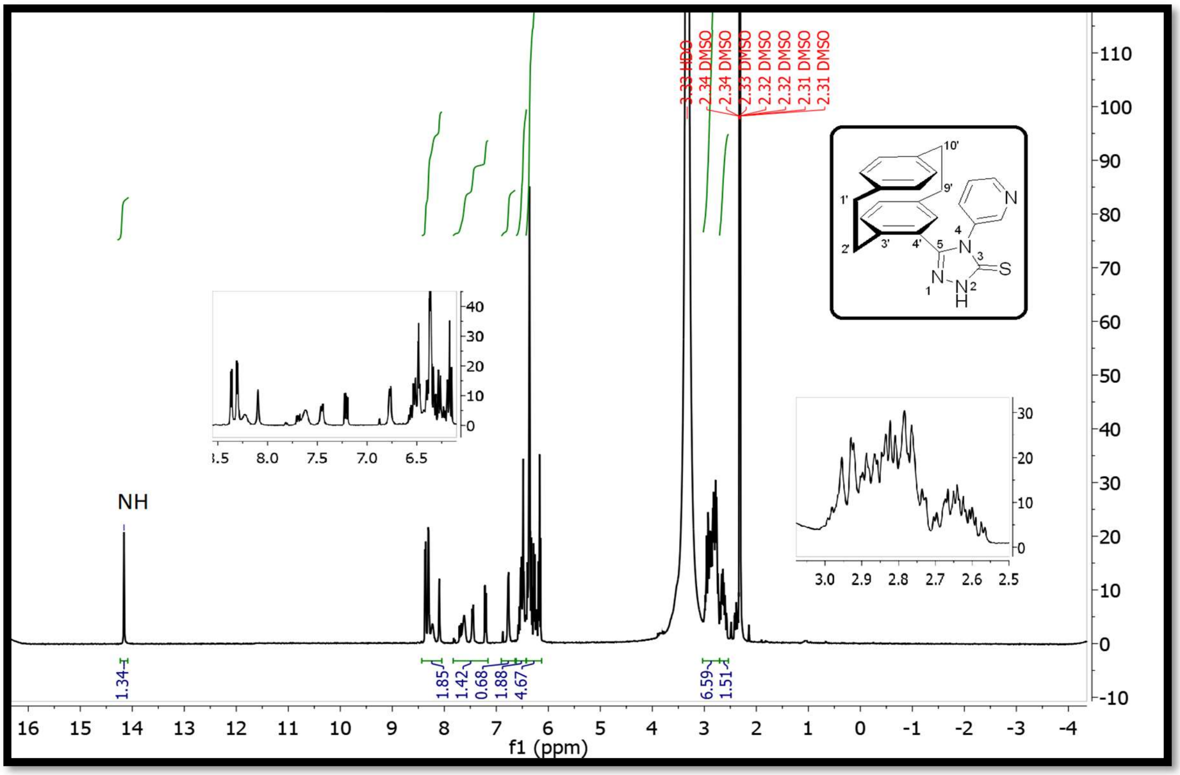

Figure 9. ^{1}H NMR of compound **12b**

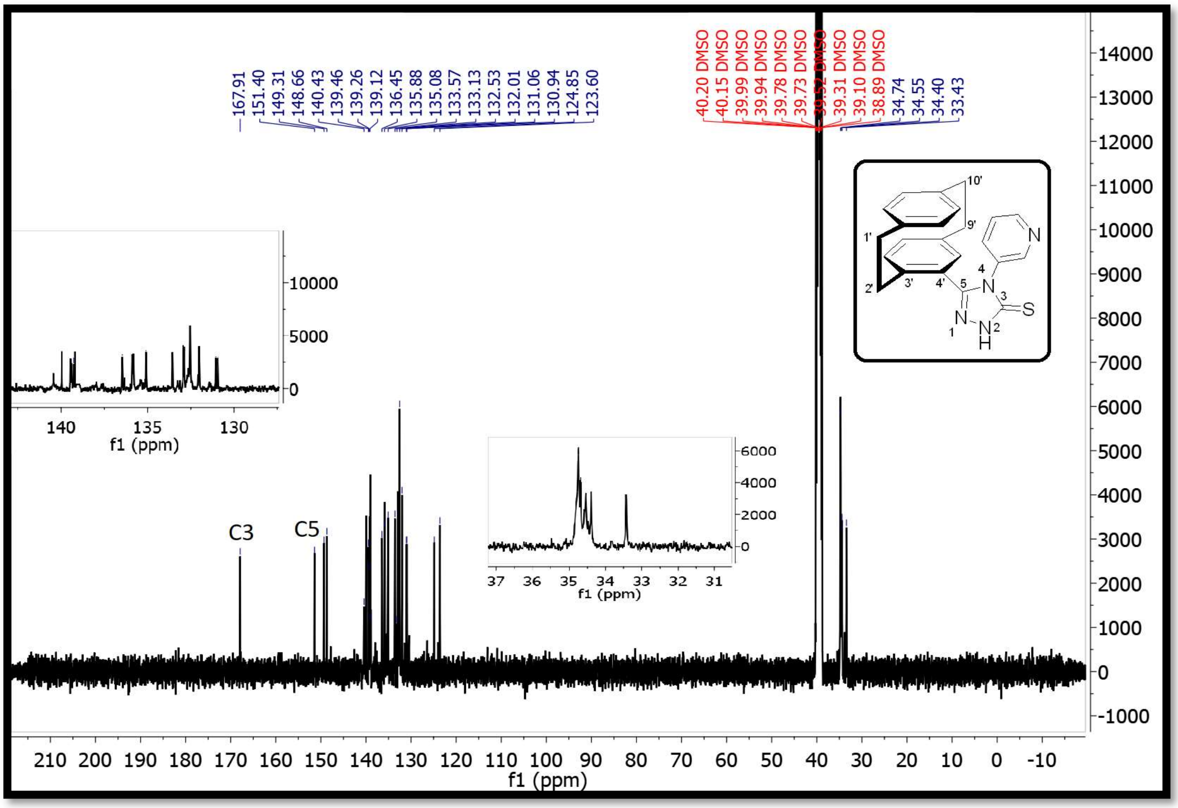

SI Figure 10. ^{13}C NMR of compound **12b**

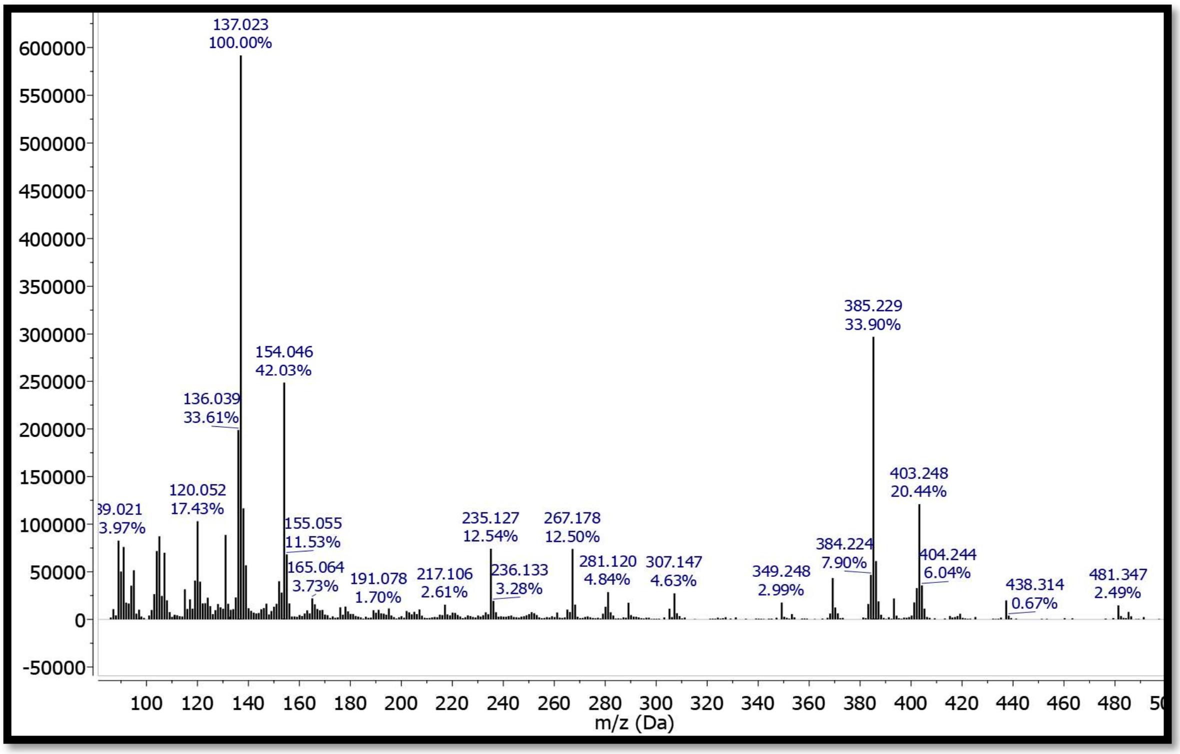

SI Figure 11. Mass spectrum of compound **12b**

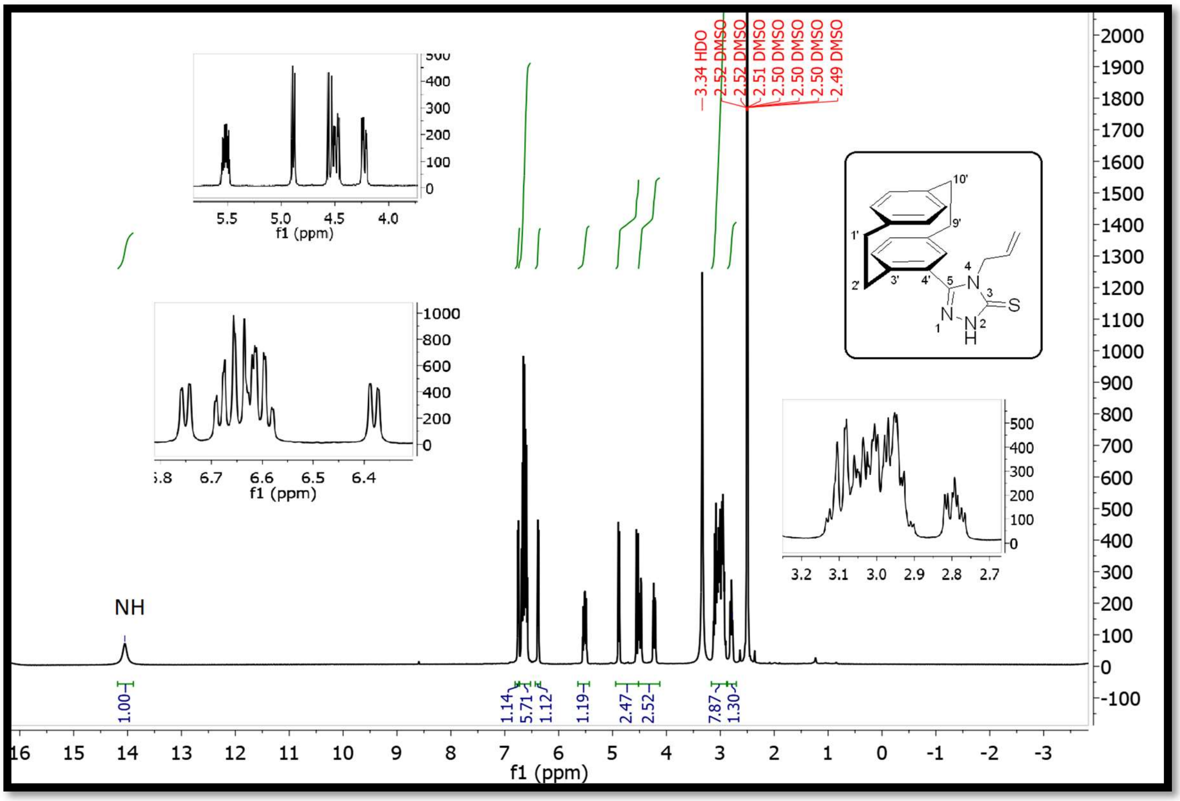

SI Figure 12. ^{1}H NMR spectrum of compound **12c**

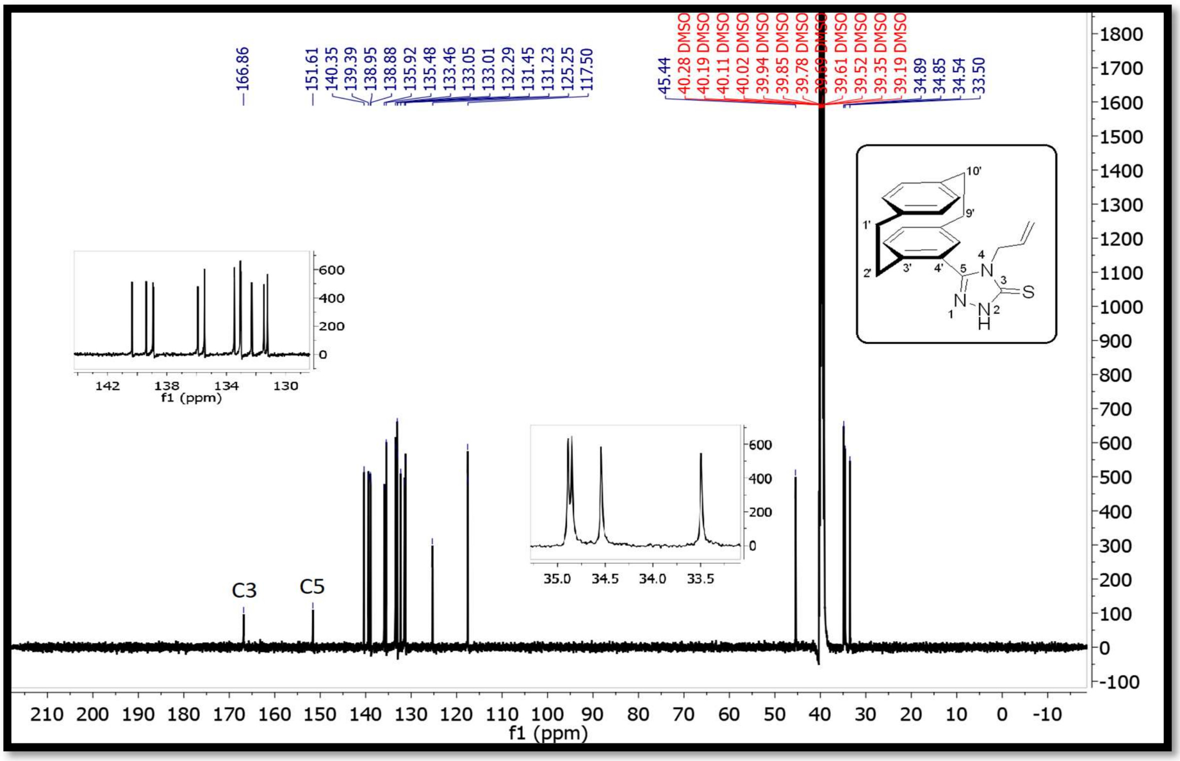

SI Figure 13. ^{13}C NMR spectrum of compound **12c**

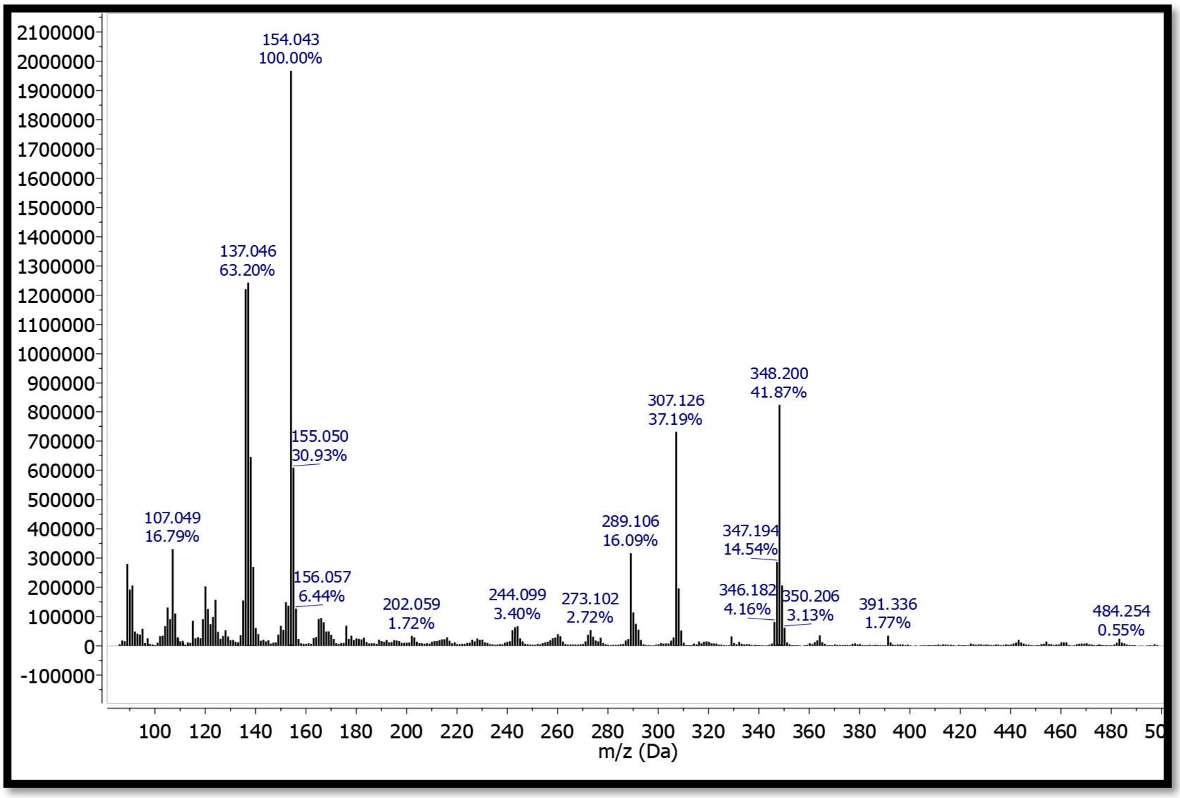

SI Figure 14. Mass spectrum of compound **12c**

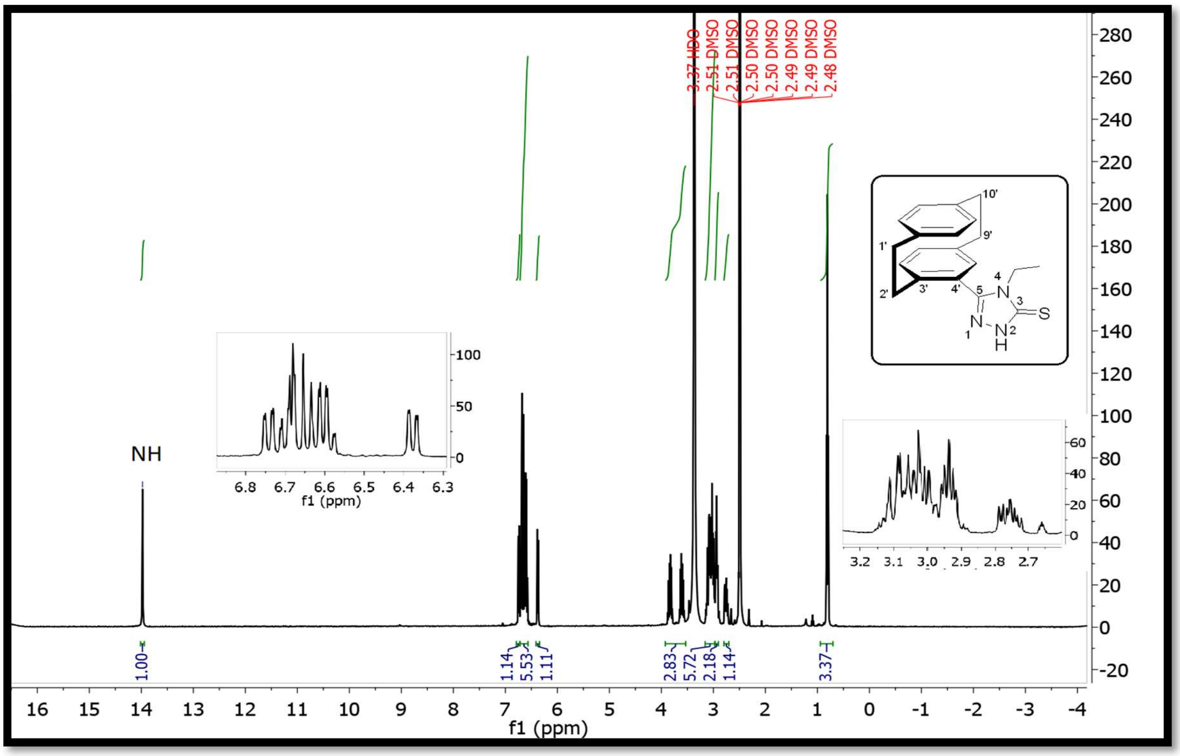

SI Figure 15. ^{1}H NMR spectrum of compound **12d**

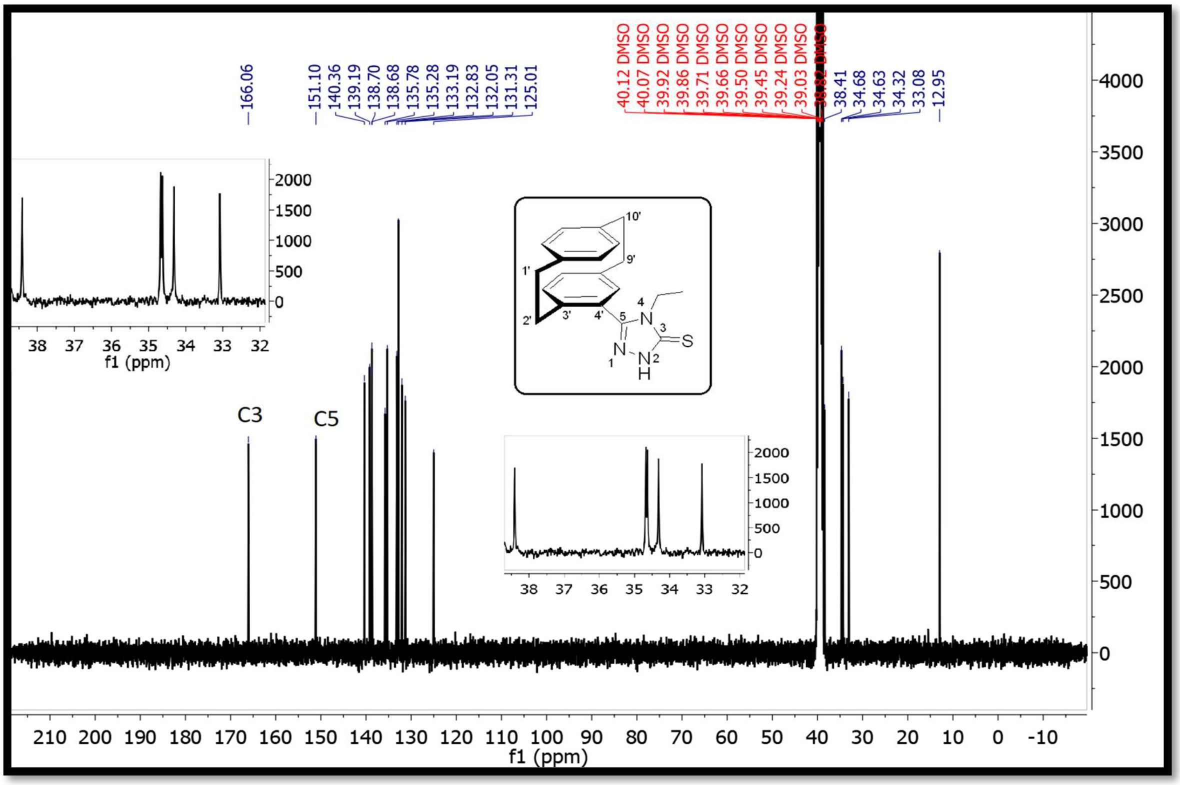

SI Figure 16. ^{13}C NMR spectrum of compound **12d**

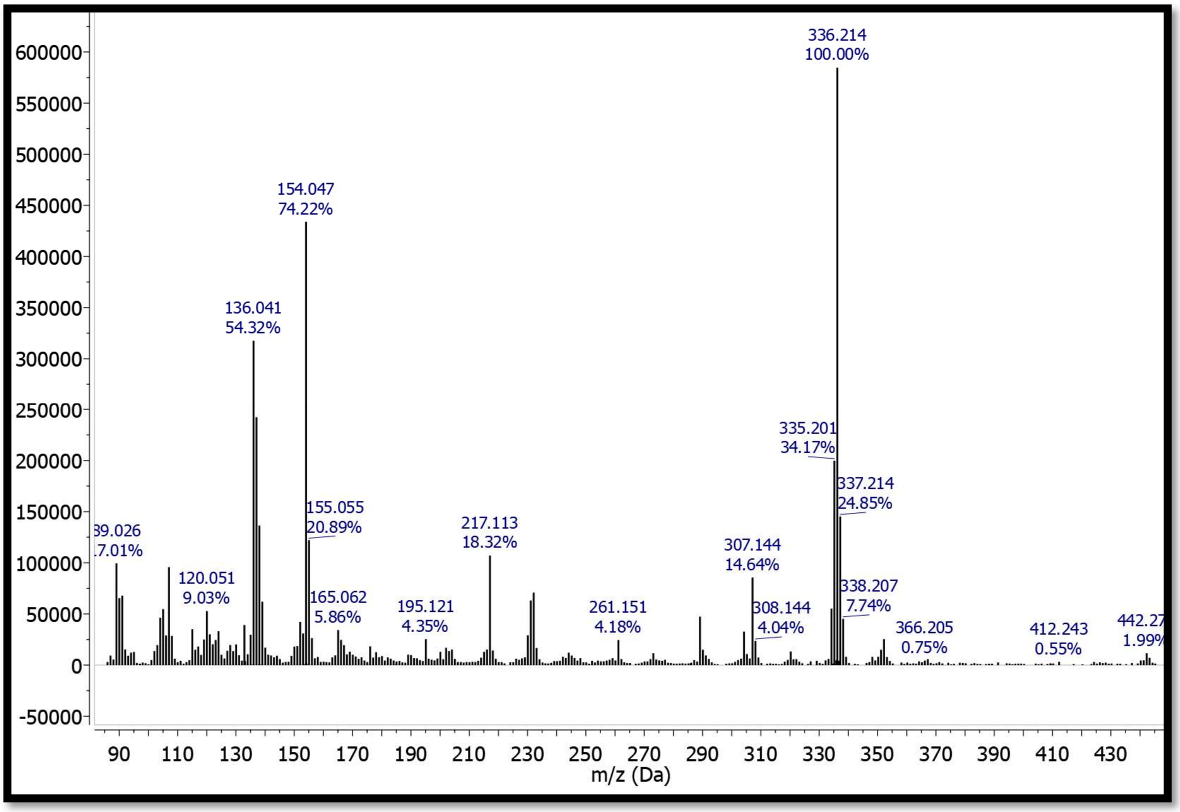

SI Figure 17. Mass spectrum of compound **12d**

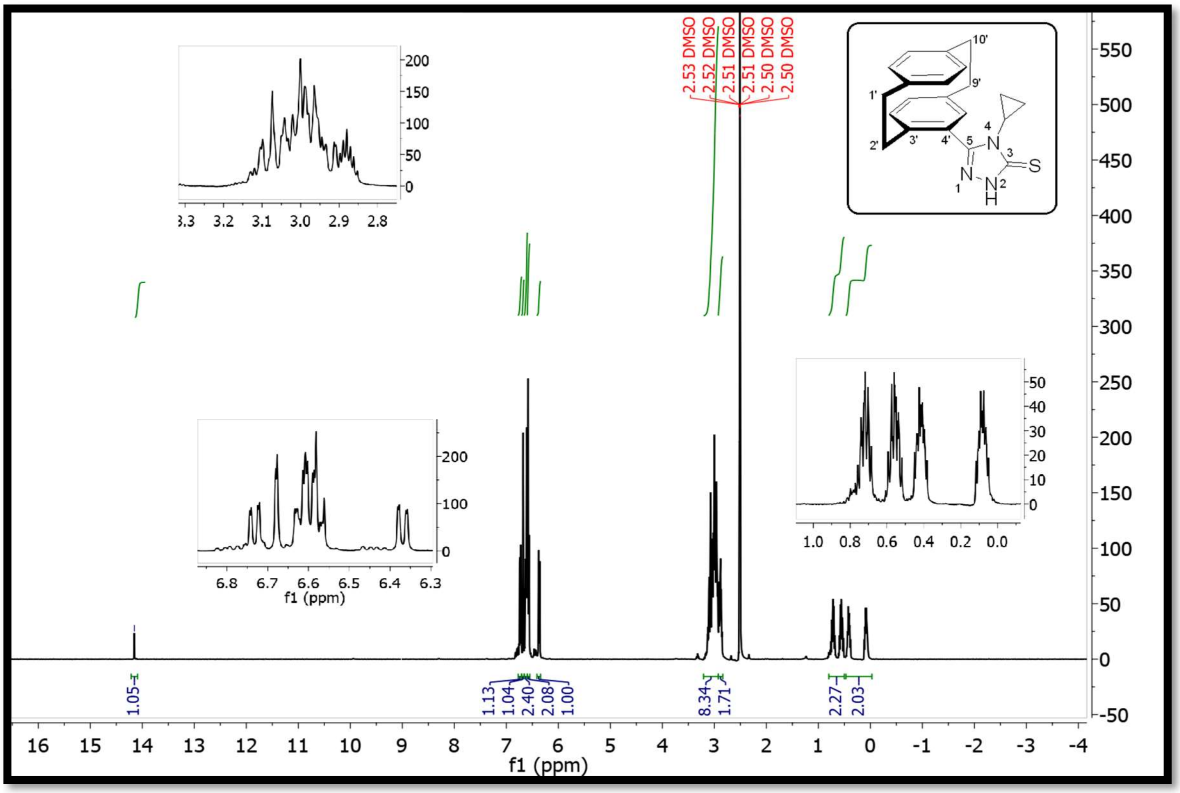

SI Figure 18. ^{1}H NMR spectrum of compound **12e**

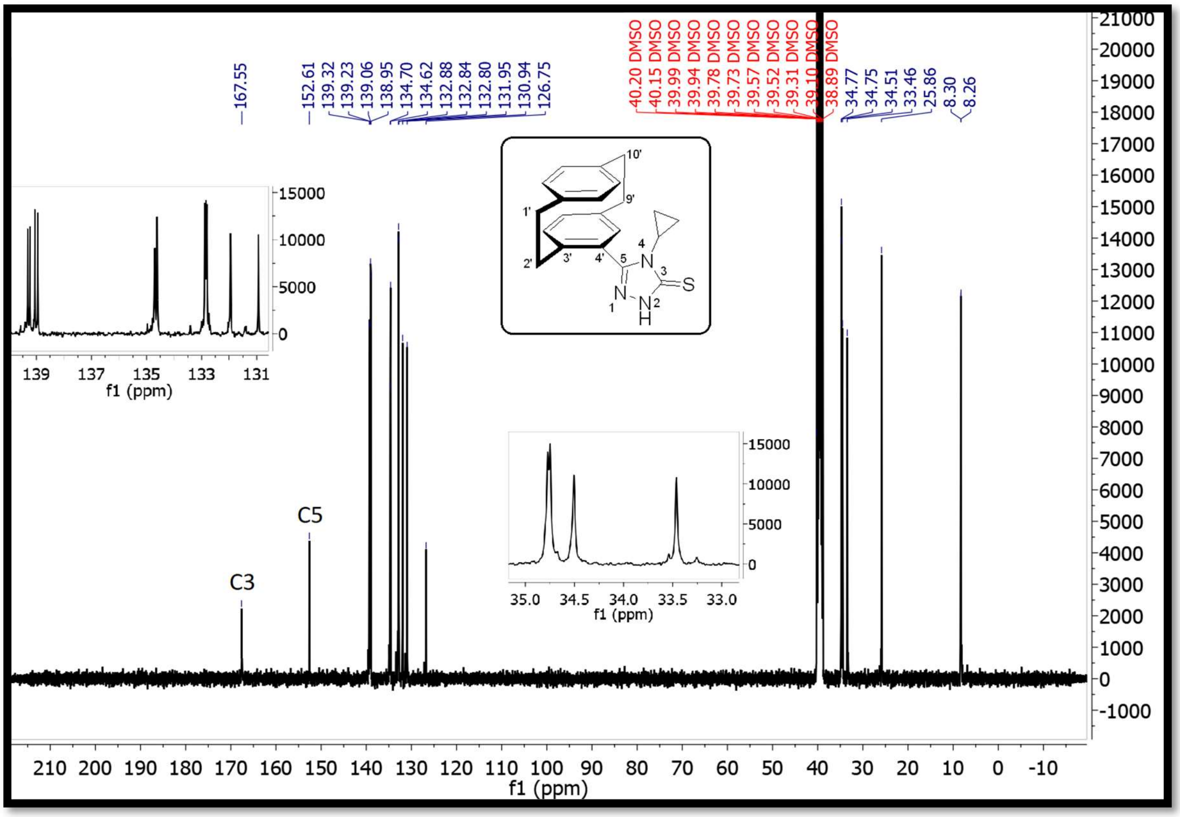

SI Figure 19. ^{13}C NMR spectrum of compound **12e**

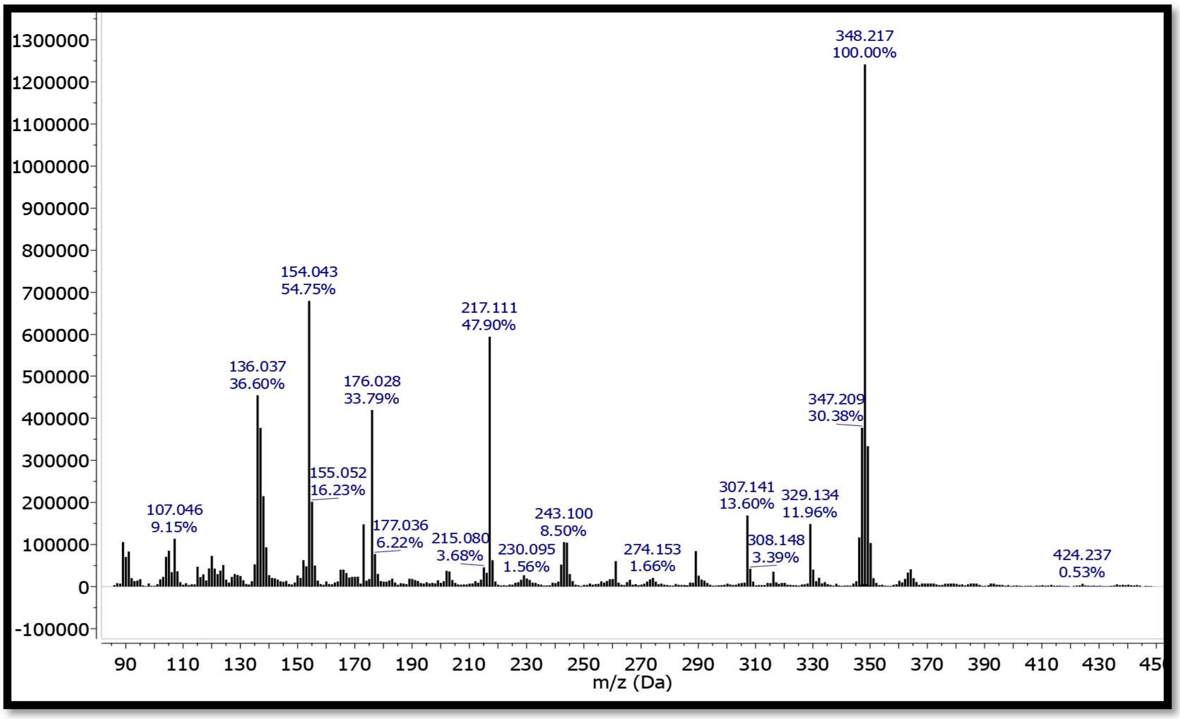

SI Figure 20. Mass spectrum of compound **12e**

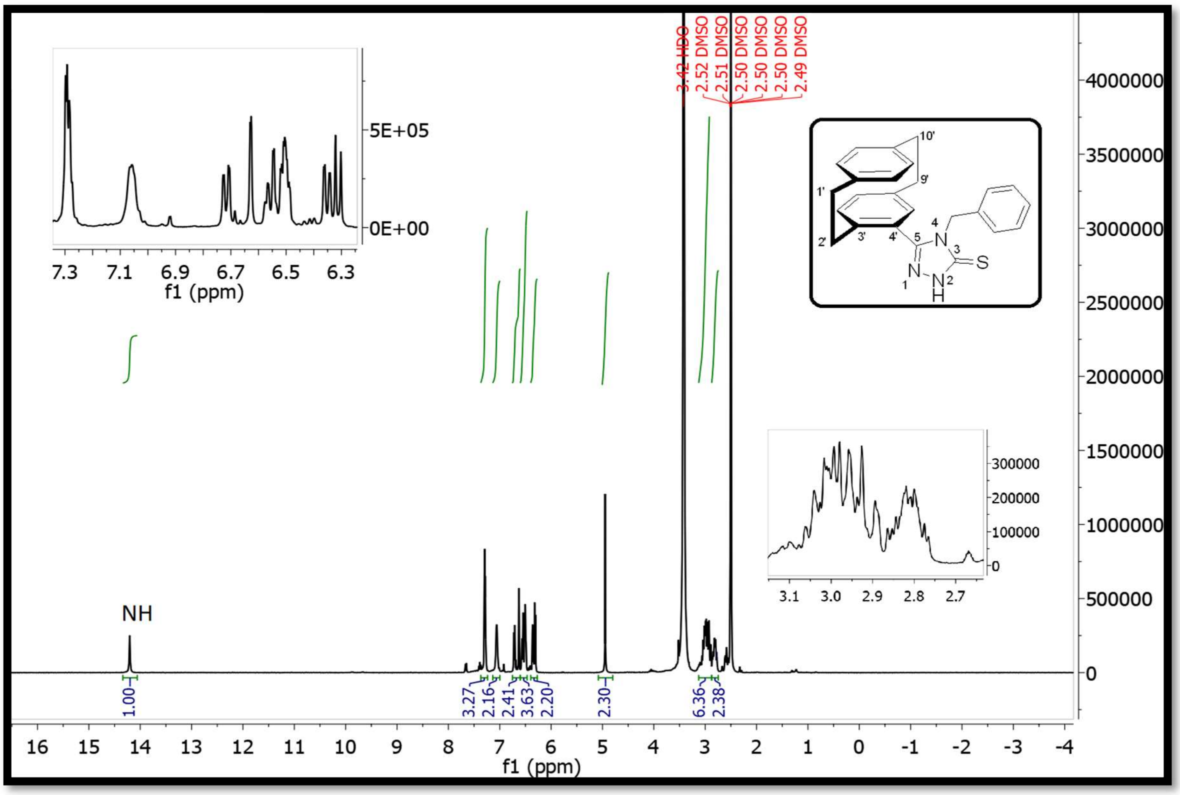

SI Figure 21. ^{1}H NMR spectrum of compound **12f**

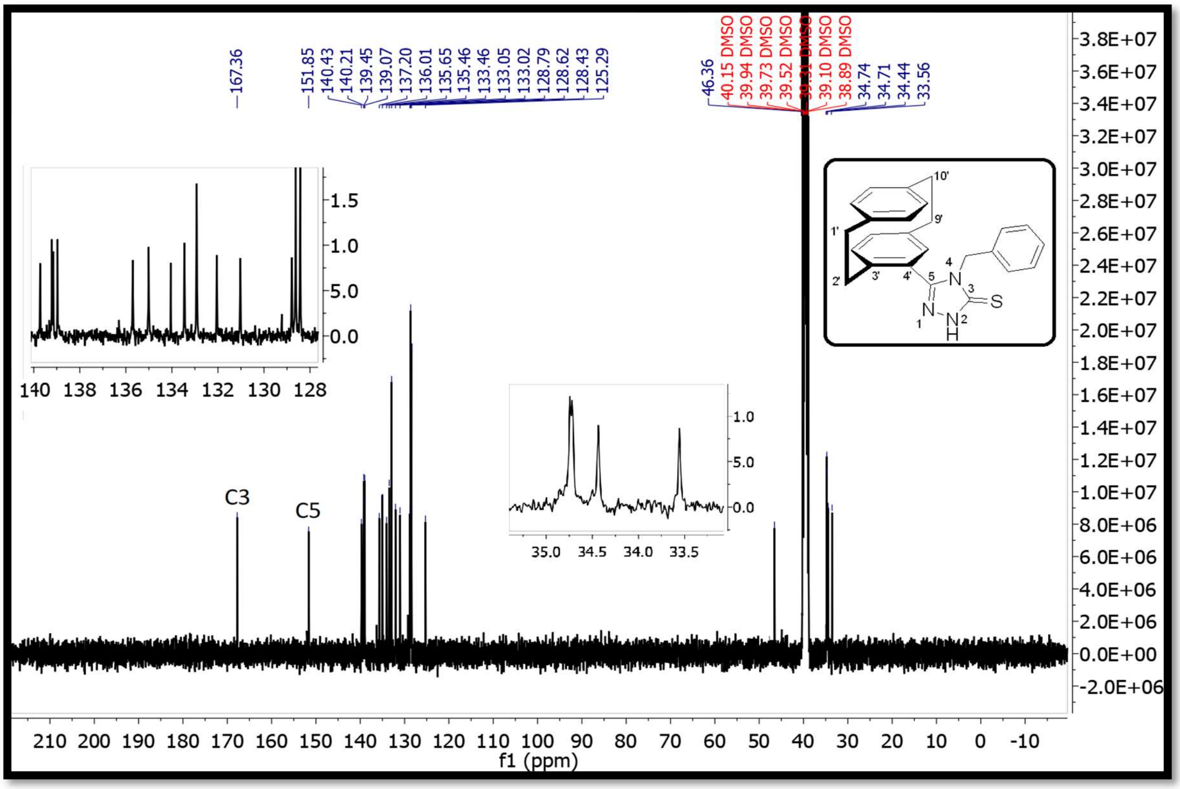

SI Figure 22. ^{13}C NMR spectrum of compound **12f**

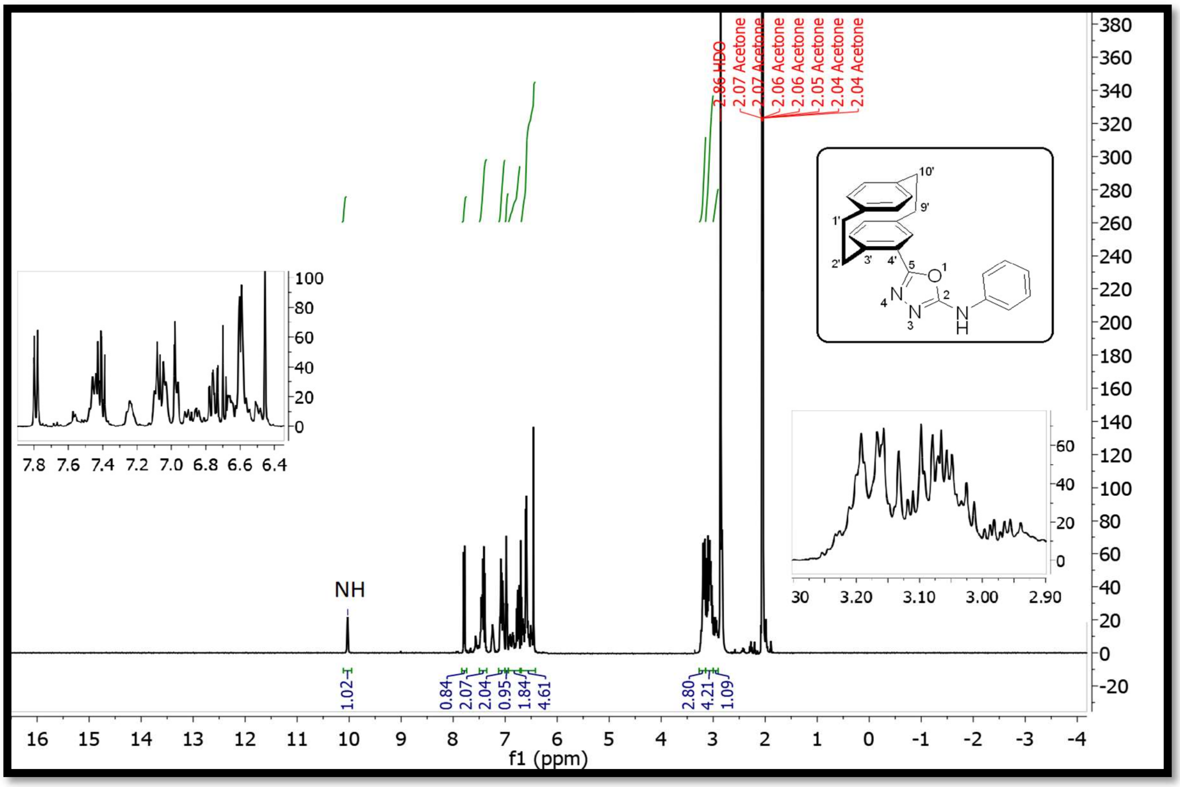

SI Figure 23. ^{1}H NMR spectrum of compound **13a**

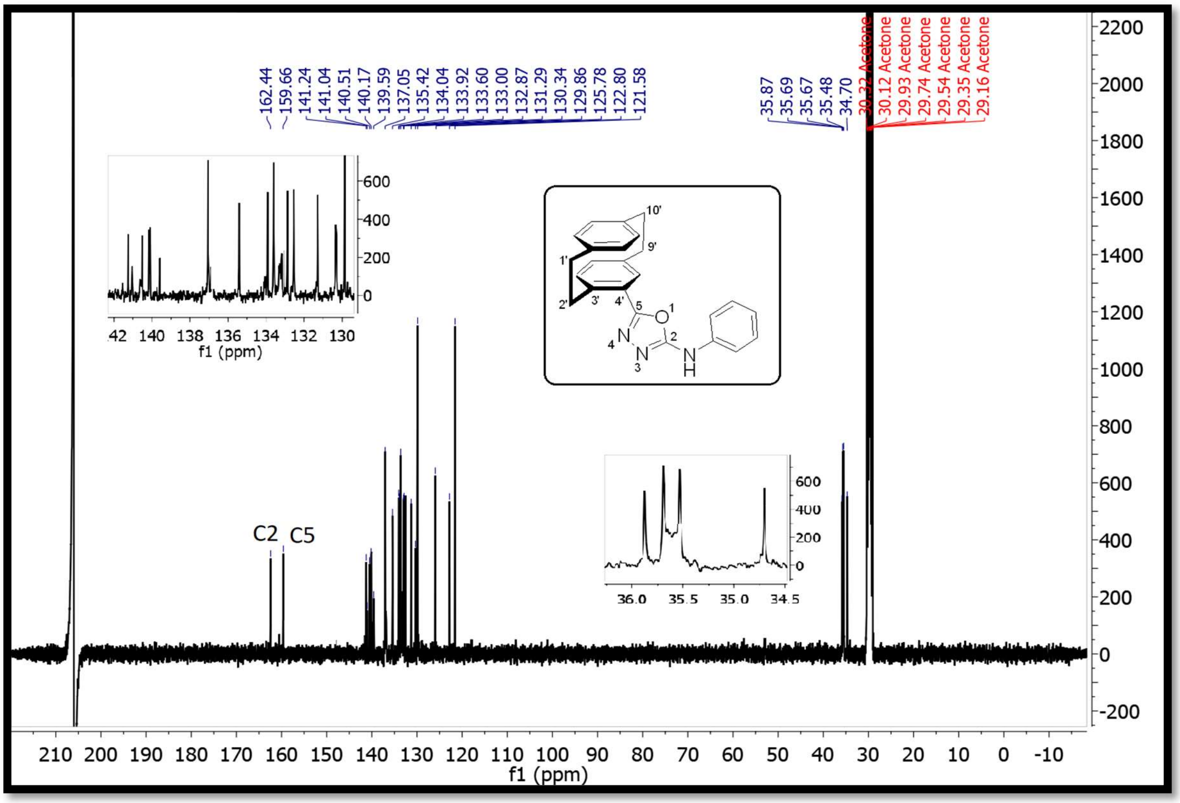

SI Figure 24. ^{13}C NMR spectrum of compound **13a**

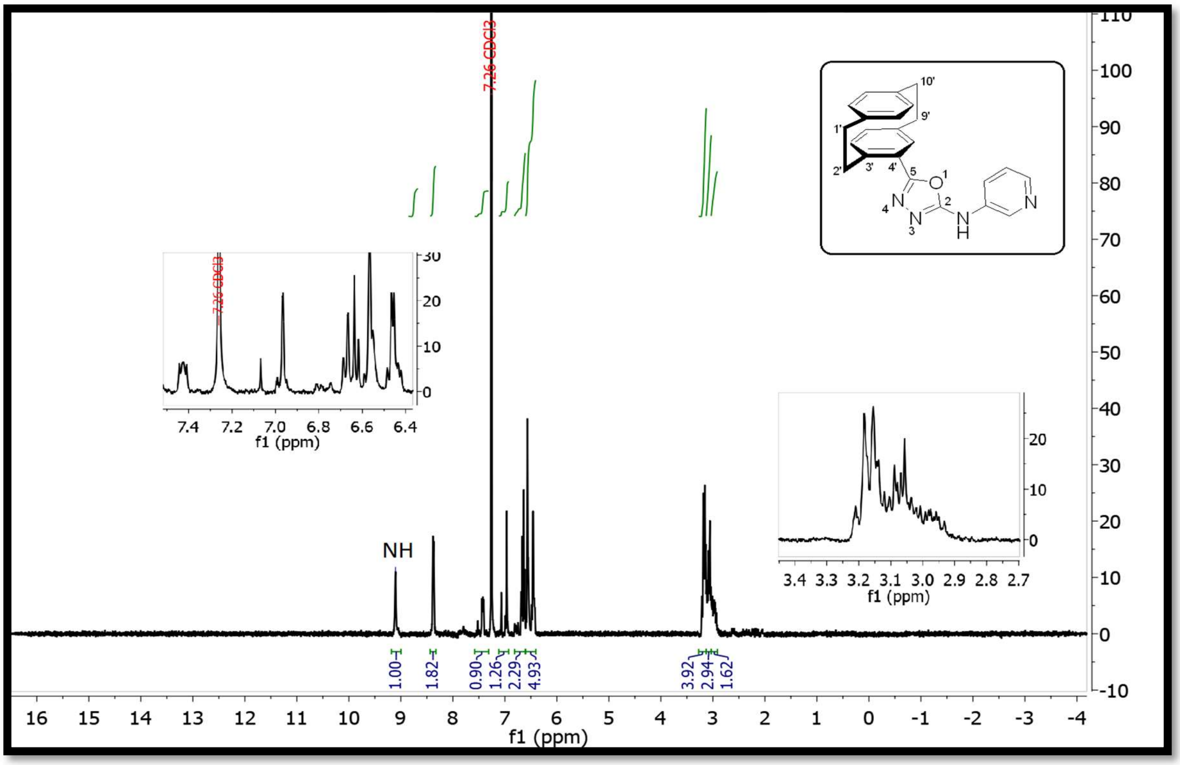

SI Figure 25. ^{1}H NMR spectrum of compound **13b**

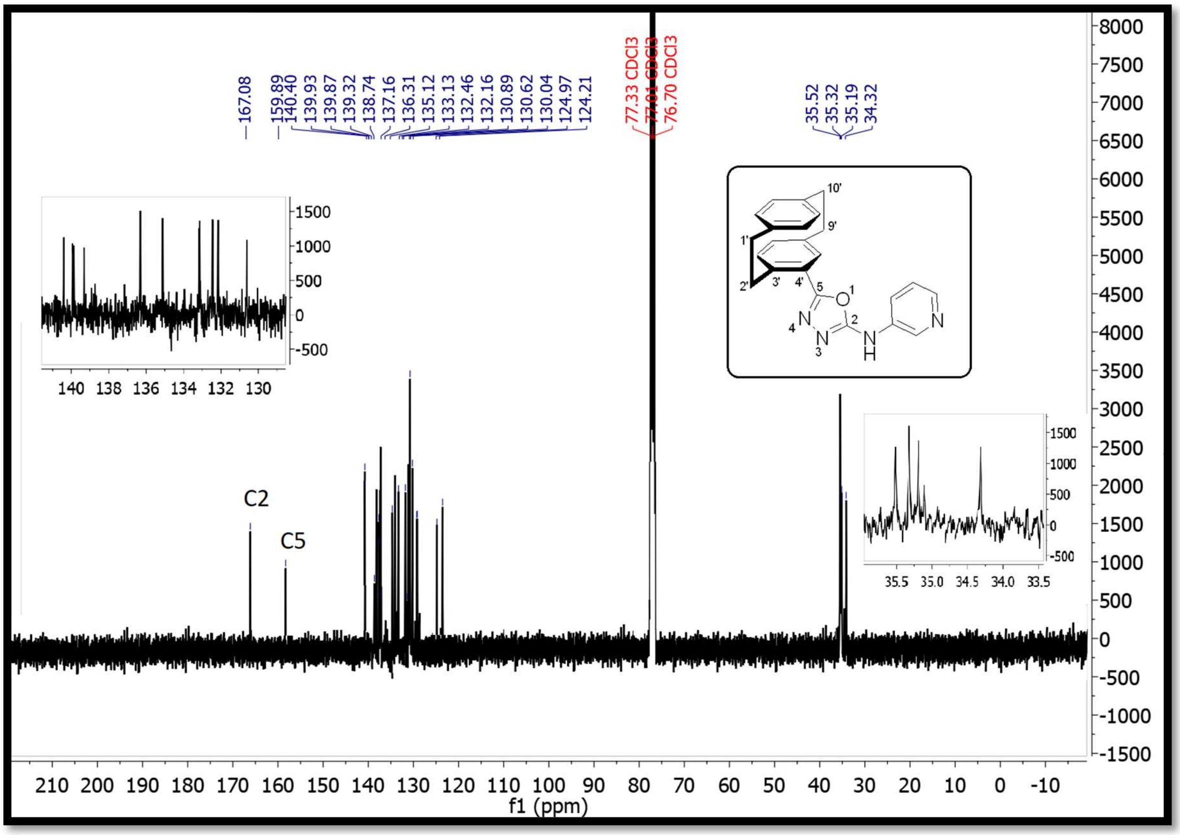

SI Figure 26. ^{13}C NMR spectrum of compound **13b**

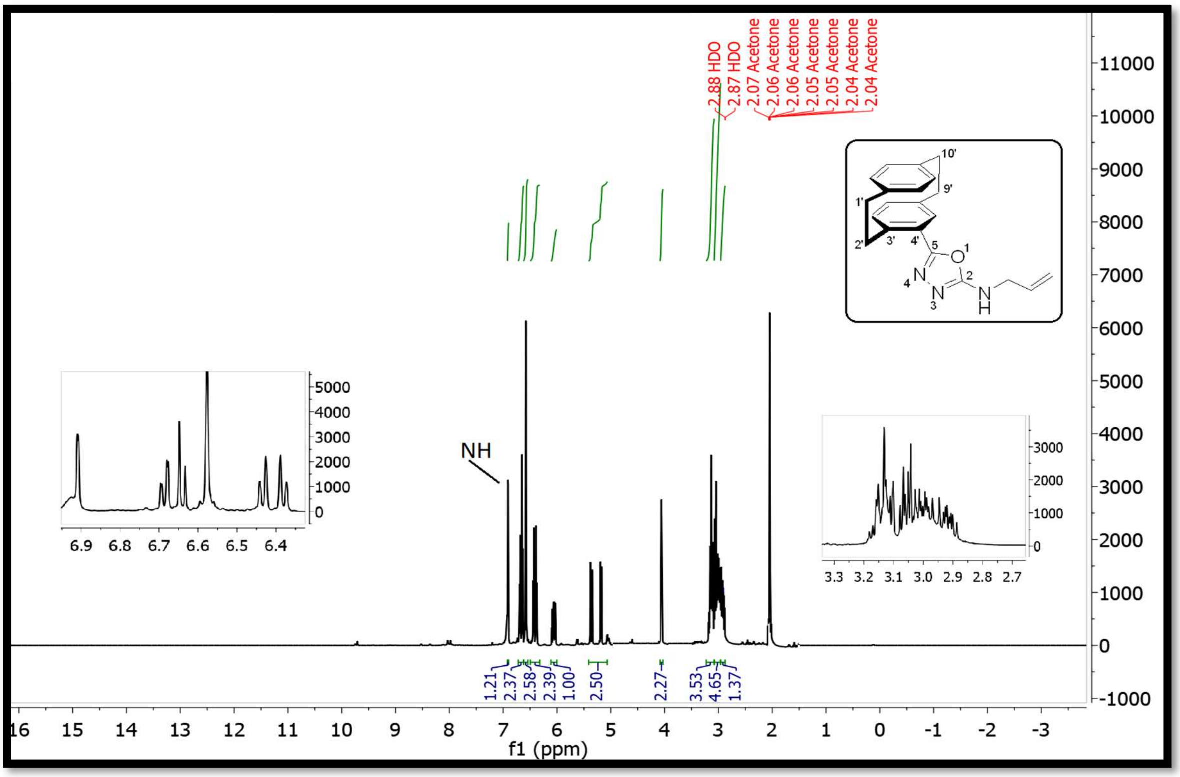

SI Figure 27. ^{1}H NMR spectrum of compound **13c**

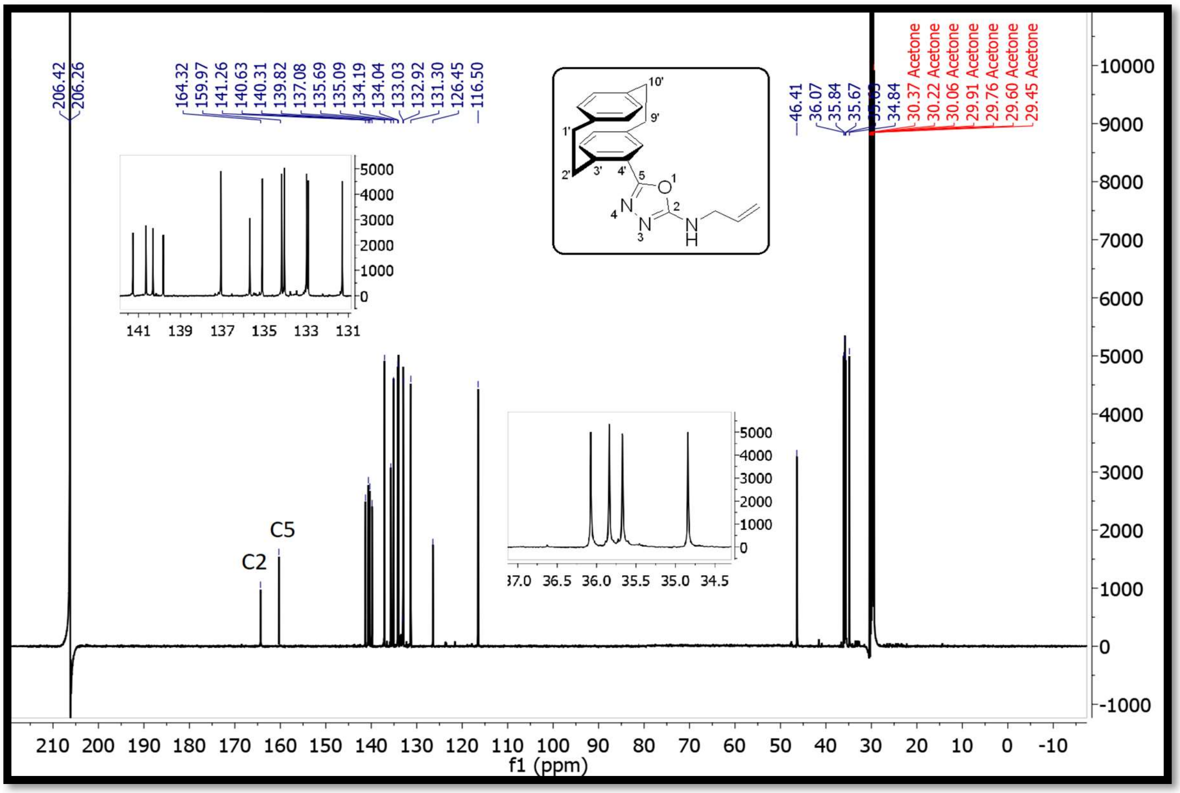

SI Figure 28. ^{13}C NMR spectrum of compound **13c**

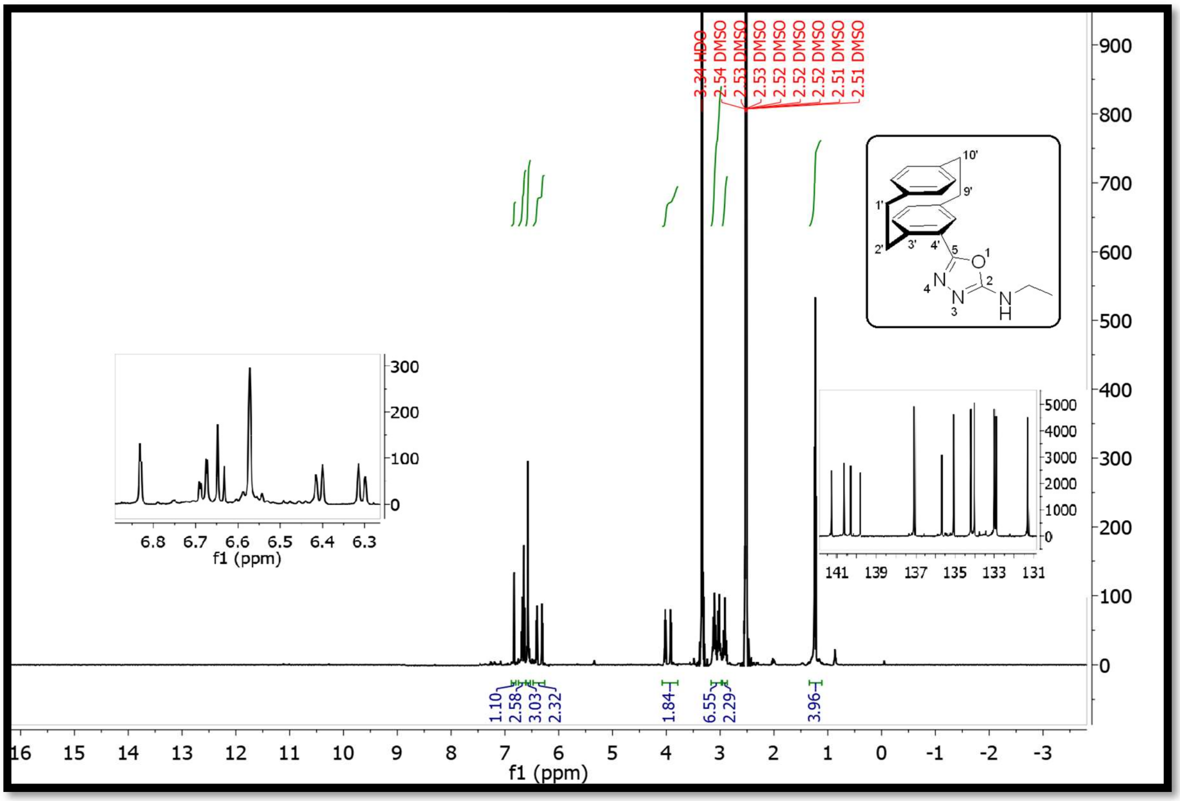

SI Figure 29. ^{1}H NMR spectrum of compound **13d**

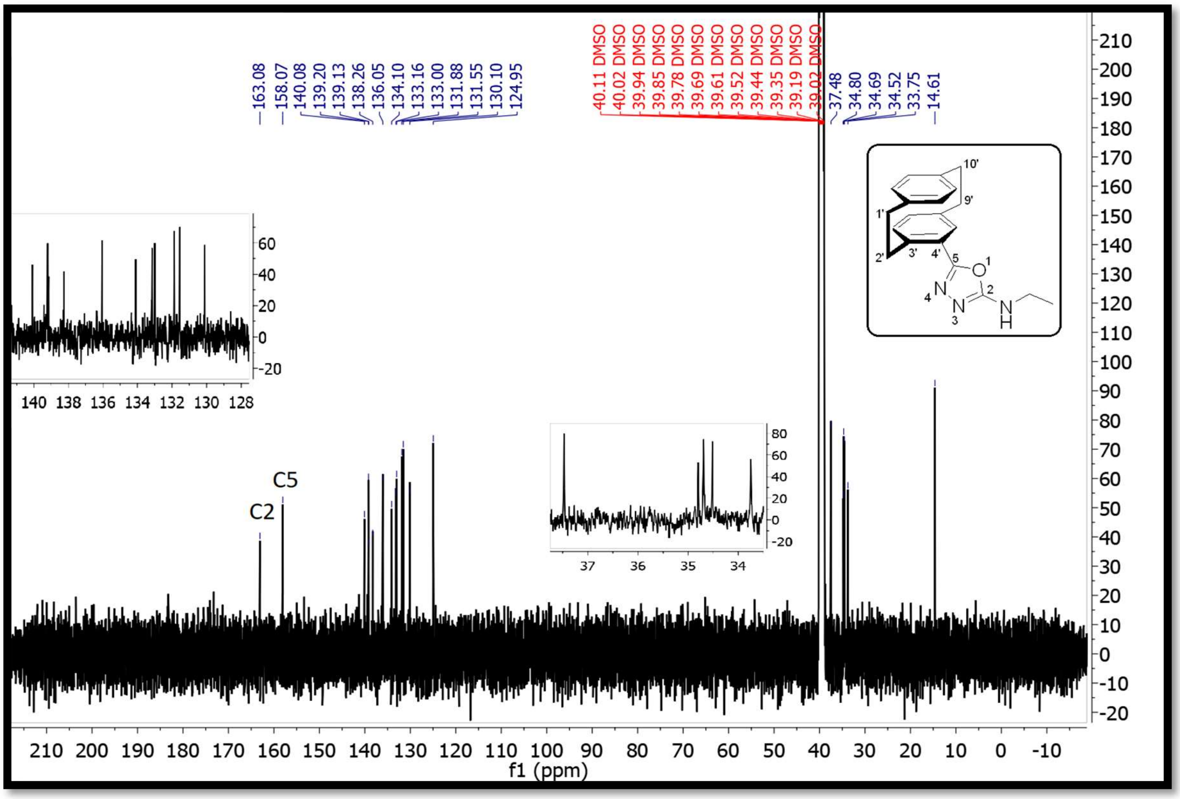

SI Figure 30. ^{13}C NMR spectrum of compound **13d**

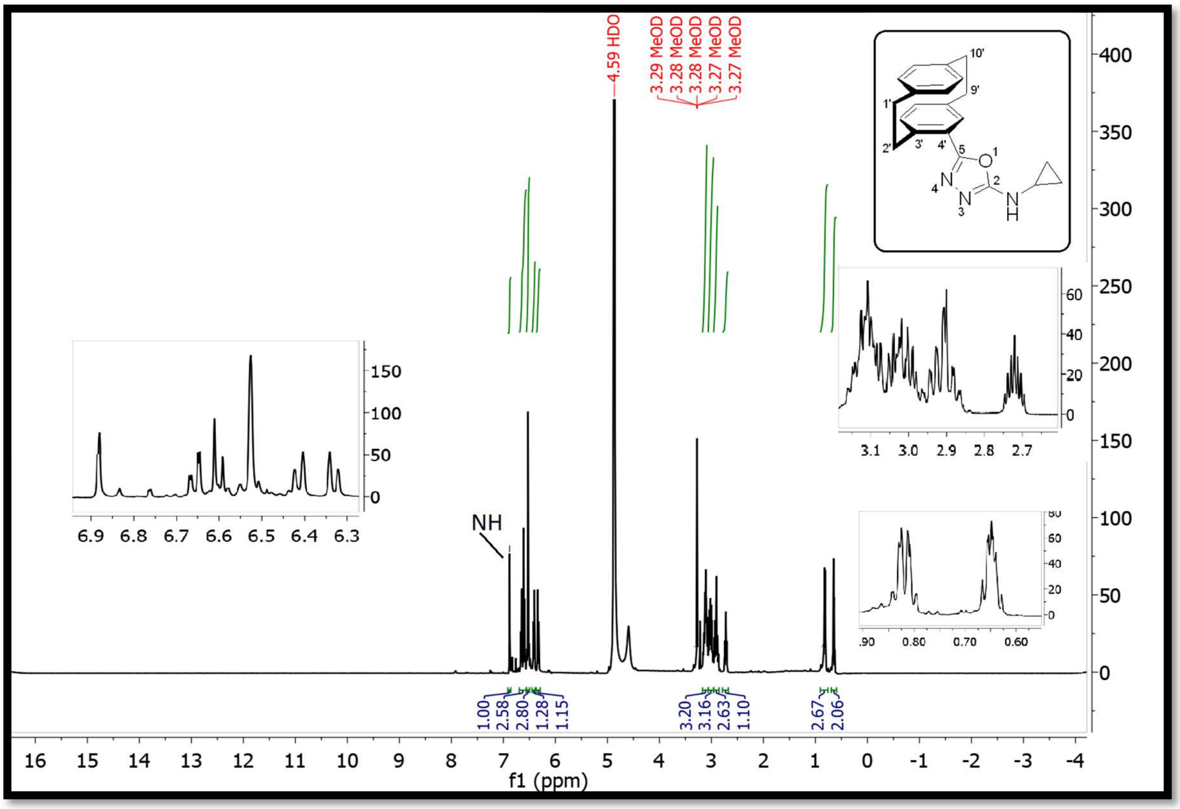

SI Figure 31. ^{1}H NMR spectrum of compound **13e**

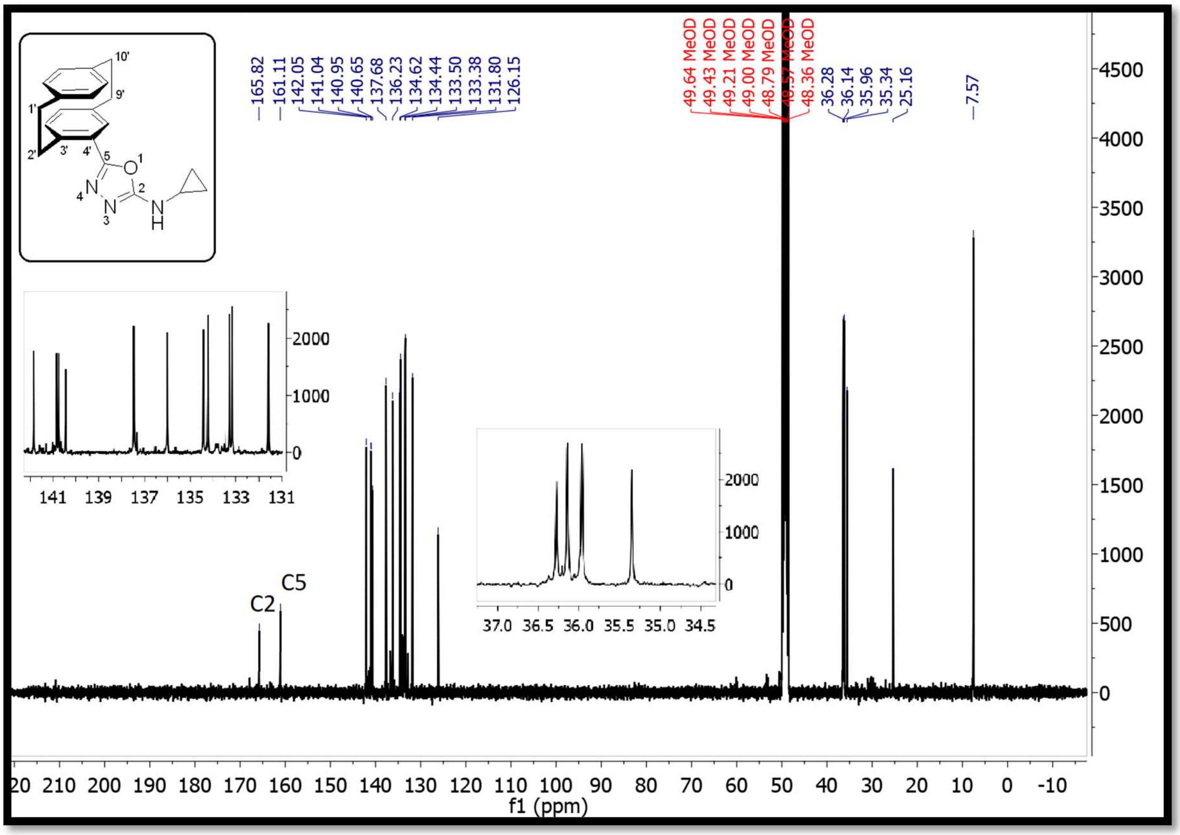

SI Figure 32. ^{13}C NMR spectrum of compound **13e**
